# Single-Cell Transcriptome Identifies the Renal Cell Type Tropism of Human BK Polyomavirus

**DOI:** 10.3390/ijms24021330

**Published:** 2023-01-10

**Authors:** Feng Yang, Xutao Chen, Hui Zhang, Guo-Dong Zhao, Huifei Yang, Jiang Qiu, Siyan Meng, Penghan Wu, Liang Tao, Qin Wang, Gang Huang

**Affiliations:** 1 Organ Transplant Center, The First Affiliated Hospital of Sun Yat-Sen University, Guangzhou 510080, China; 2 Department of Pharmacology, Zhongshan School of Medicine, Sun Yat-Sen University, Guangzhou 510080, China; 3 Guangdong Provincial Key Laboratory of Organ Donation and Transplant Immunology, Sun Yat-Sen University, Guangzhou 510080, China; 4 Guangdong Provincial International Cooperation Based of Science and Technology (Organ Transplantation), Sun Yat-Sen University, Guangzhou 510080, China; 5 Department of Pharmacology, Guangzhou University of Chinese Medicine, Guangzhou 510080, China

**Keywords:** BK polyomavirus, single-cell transcriptomics, tropism, immune microenvironment, metabolism, multi-omics

## Abstract

BK polyomavirus (BKPyV) infection is the main factor affecting the prognosis of kidney transplant recipients, as no antiviral agent is yet available. A better understanding of the renal-cell-type tropism of BKPyV can serve to develop new treatment strategies. In this study, the single-cell transcriptomic analysis demonstrated that the ranking of BKPyV tropism for the kidney was proximal tubule cells (PT), collecting duct cells (CD), and glomerular endothelial cells (GEC) according to the signature of renal cell type and immune microenvironment. In normal kidneys, we found that BKPyV infection-related transcription factors P65 and CEBPB were PT-specific transcription factors, and PT showed higher glycolysis/gluconeogenesis activities than CD and GEC. Furthermore, in the BKPyV-infected kidneys, the percentage of late viral transcripts in PT was significantly higher than in CD and GEC. In addition, PT had the smallest cell–cell interactions with immune cells compared to CD and GEC in both normal and BKPyV-infected kidneys. Subsequently, we indirectly demonstrated the ranking of BKPyV tropism via the clinical observation of sequential biopsies. Together, our results provided in-depth insights into the renal cell-type tropism of BKPyV in vivo at single-cell resolution and proposed a novel antiviral target.

## 1. Introduction

BKPyV infection starts with the attachment of VP1 to host cellular receptors, an N-linked glycoprotein or ganglioside GD1b or GT1b, all of which contain an α (2,3)-linked sialic acid residue [1,2]. Following initial attachment to the cell surface, BKPyV is internalized into the target cell via a caveola-mediated endocytic pathway [3,4]. BKPyV was subsequently trafficked to the endoplasmic reticulum along the microtubule network, from which it follows the endoplasmic reticulum associated protein degradation for capsid uncoating [5,6,7]. The viral genome is then transported into the nucleus via the nuclear pore complex thanks to VP2/VP3, nuclear localization signal, and the importin α/β1 import pathway [8]. Once in the cell nucleus, the BKPyV genome is transcribed. The newly synthesized viral DNA is packaged to form capsids [9]. Progeny virions are mainly released from infected cells after cell lysis [9,10]. With the introduction of more potent immunosuppression regimens, BK polyomavirus (BKPyV) infection has become an increasing cause of allograft failure in kidney transplant recipients (KTRs) [11,12]. BKPyV is highly prevalent among KTRs with viruria, viremia, and BKPyV-associated nephropathy (BKPyVAN) with rates of 30–50%, 10–15%, and 1–10%, respectively [13]. Despite its high prevalence, there is no FDA-approved BKPyV-specific antiviral drug, and clinical management of BKPyV infection is customary to reduce immunosuppressants, which is not always curative and puts the allograft at risk for rejection [14].

Virus tropism is an important determinant of disease progression associated with virus infection [15,16]. Therefore, exploring the renal cell type tropism of BKPyV might help to develop effective treatment options for BKPyVAN. Based on previous studies [17,18], we thought two main factors affected the BKPyV tropism: cell type signatures and the immune microenvironment. First, BKPyV utilizes host cell machinery and modulates host cell transcriptome for replication or evading host immune responses. Therefore, cell-type signatures determine whether infection can be successfully established within a specific cell type [17]. Second, BKPyV only affects immunocompromised individuals. Therefore, host immunity against BKPyV is critical in dictating viral tropism [18]. However, current in vitro studies on BKPyV tropism do not entirely mimic the in vivo immune microenvironment of the kidney [19,20]. Additionally, BKPyV displays a very narrow host range that precludes direct investigation of the pathogenesis of BKPyV infection in animal models [21]. Thus, the renal cell type tropism of BKPyV is still unclear.

The recently available large-scale single-cell RNA-sequencing (scRNA-seq) can quantify gene expression at single-cell resolution in vivo [22,23]. Therefore, scRNA-seq provides an unbiased tool to investigate the molecular characterization of a single cell and the interplay between renal cells and immune cells. In this study, our objective was to provide new insights into the cell type preference of BKPyV in the kidneys using scRNA-seq and explore potential therapeutic targets to prevent BKPyV infection based on multi-omics.

## 2. Results

### 2.1. Identification of Major Cell Types in Normal Kidneys

**Figure 1:**
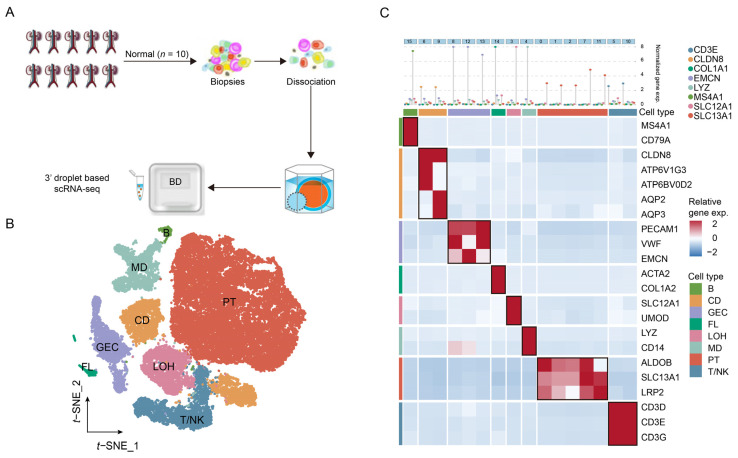
Cell type identification in normal kidneys. (**A**) Overview of sample collection and single-cell transcriptomic analysis. (**B**) The t−SNE plot of 46,287 cells in the normal adult kidney from 10 samples color-coded by the major cell types as indicated. (**C**) Two−layered complex heatmap of selected canonical markers in each cell type. Top: mean expression of canonical markers. Bottom: relative expression map of known marker genes associated with each cell type. Relative expression values are scaled using mean−centering and transformed to a scale from −2 to 2. PT: proximal tubule cells, LOH: loop of Henle cells, CD: collecting duct cells, GEC: glomerular endothelial cells, B: B cells, T/NK: T and natural killer cells, MD: myeloid cells.

After quality control (cells with >500 genes and <4000 genes; and <30% of mitochondrial gene expression in UMI counts) and doublet removal, a total of 46,287 high-quality cells were further analyzed in normal kidneys. The average number of cells per sample was 3857, which met the needs for a follow-up analysis based on the statistical power analysis (Supplementary Figure S1 and Table S1). To increase the accuracy of cell type designation, we applied canonical correlation analysis before cell-type identification. We obtained scRNA-seq data from 10 normal samples (Figure 1A) to elucidate the cellular landscape of normal kidneys. Then, we identified eight major cell types of normal kidneys using marker-based annotations (Figure 1B): proximal tubule cells (PT, SLC13A1+); loop of Henle cells (LOH, SLC12A1+); collecting duct cells (CD, CLDN8+); glomerular endothelial cells (GEC, EMCN+); fibroblast cells (FL, COL1A1+); B cells (B, MS4A1+); T and natural killer cells (T/NK, CD3E+); and myeloid cells (MD, LYZ+) (Figure 1C, top). To achieve more accurate and efficient results, we also characterized the cell types by the co-expression of other contractile genes described in previous studies (Figure 1C, bottom). Together, we identified the major cell types in normal kidneys to further explore the BKPyV tropism in vivo.

### 2.2. Quantifying the Cell-Type Specificity of Transcription Factor (TF) in Normal Kidneys

**Figure 2:**
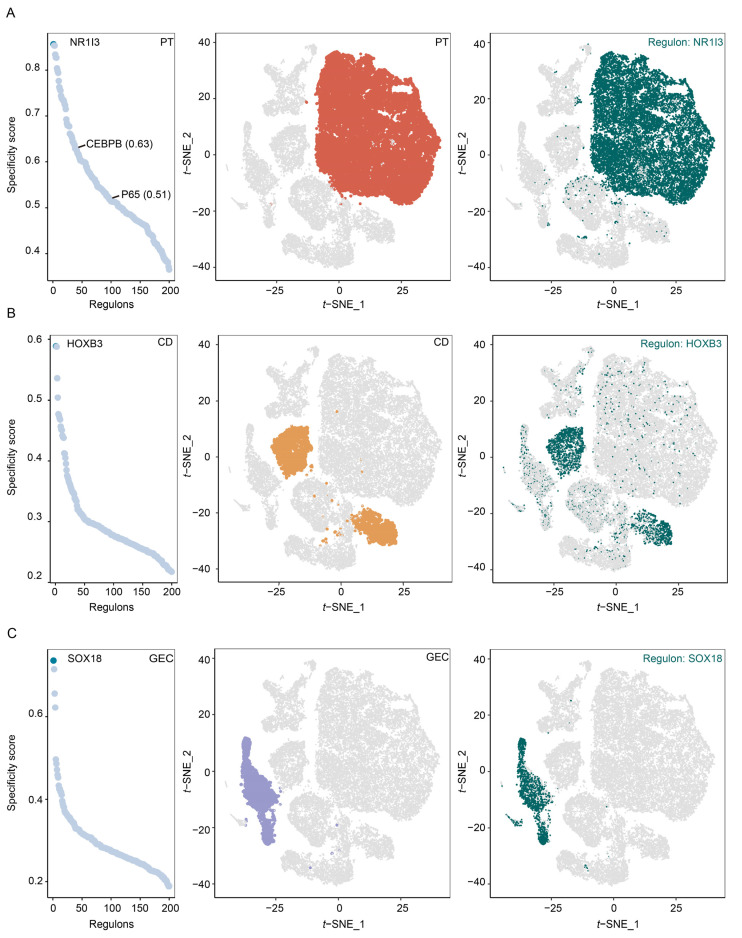
Analysis of the activity of cell type-specific transcription factors in normal kidneys. Rank for transcription factor (TF) in each cell type based on gene−TF regulatory networks specificity score (**A**–**C**, left). PT, CD, and GEC were represented in the t-SNE plots (**A**–**C**, middle). The color indicated cell type. Binarized TF activity scores for a listed first TF of each cell type on the t-SNE plot (**A**–**C**, right). PT: proximal tubule cells, CD: collecting duct cells, GEC: glomerular endothelial cells.

We compared the BKPyV tropism among CD, PT, and GEC in normal kidneys based on the perspective of cell type signatures. According to a previous study [17], TF played a crucial role in maintaining the cell type signatures and could regulate the expression of BKPyV. Thus, we identified which kind of cell was suited for BKPyV replication using cell type-specific TFs. Single-Cell Regulatory Network Inference and Clustering (SCENIC) analysis systematically identified critical TFs for PT, CD, and GEC. We evaluated each TF’s activities associated with each cell type and defined the gene-TF regulatory networks (regulons) specificity score (RSS) based on the Jensen–Shannon divergence (Supplementary Table S2). Then, we identified NR1I3, HOXB3, and SOX18 as the most specific TF associated with PT, CD, and GEC, respectively (Figure 2A–C, left). tSNE plots provided additional support that the activities of the TF were highly specific to PT, CD, and GEC, respectively (Figure 2A–C, middle and right). Of note, P65 (also known as RELA or NFKB3) and CEBPB, which were reported to regulate BKPyV gene expression [24,25], were found as the specific TFs for PT based on the RSS (0.51 and 0.63 > 0.5, respectively). Together, we showed that PT were more suited for BKPyV replication than CD and GEC from the perspective of cell type-specific TFs in normal kidneys.

### 2.3. Comparison of the Activity of Glycolysis/Gluconeogenesis in Normal Kidneys

**Figure 3:**
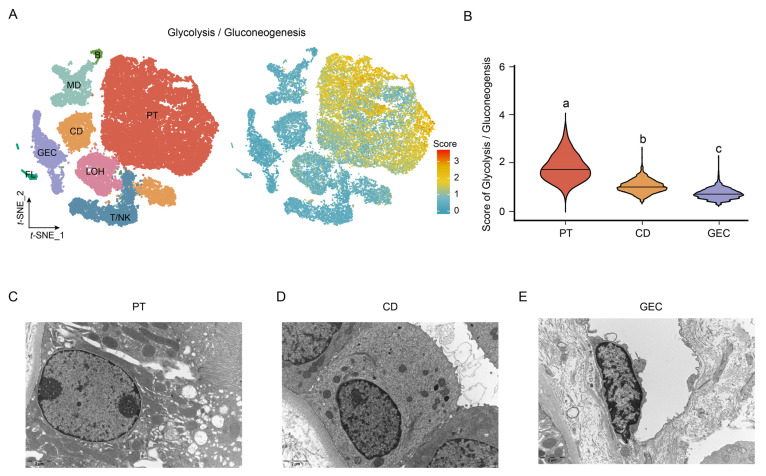
Comparison of the activity of glycolysis/gluconeogenesis among the normal renal cells. (**A**) The metabolic activity analysis of renal cells showed that PT had the highest glycolysis/gluconeogenesis metabolic activity in normal kidneys. (**B**) The violin plot showed the glycolysis/gluconeogenesis score among normal renal cells with Kruskal–Wallis H test. Significant differences among PT, CD, and GEC were denoted by different letters (*p* < 0.05). Electron microscopy pictures of PT (**C**), CD (**D**), and GEC (**E**) showed that PT had higher numbers of organelles in the cytoplasm, more villi on the cell surface, rich euchromatin, and more distinct nucleolus than the CD and GEC.

Since BKPyV utilizes host cell machinery for replication, the more active the metabolism, the more likely the BKPyV could be established within a specific cell type [17]. However, BKPyV could modulate the host cell transcriptome for reproduction. Therefore, we compared the active of cell metabolism in normal kidneys. The scMetabolism analysis showed that cells with a high glycolysis/gluconeogenesis score were clustered in PT indicating that PT were more active than CD and GEC (Figure 3A). Moreover, the violin plot showed that the ranking of glycolysis/gluconeogenesis activity score was PT > CD > GEC and different letters indicated statistically significant differences among the three groups with the Kruskal–Wallis H test (*p* < 0.05) (Figure 3B). We also evaluated other metabolic pathways. We found most of the metabolism pathways were enriched in PT compared to CD and GEC (Supplementary Figure S2). In addition, we observed the normal renal cells using transmission electron microscopy (TEM) to further verify the cell metabolic status, as the morphology of the nucleolus, chromatin state, and villi was thought to be associated with the cellular metabolic state and cell function [26,27]. PT (Figure 3C) showed higher numbers of organelles in the cytoplasm, more villi on the cell surface, richer euchromatin, and more distinct nucleolus than CD (Figure 3D) and GEC (Figure 3E). The TEM results indicated that PT showed higher metabolism activities than CD and GEC under normal conditions.

### 2.4. Complex Intercellular Communication Networks in Normal Kidneys

**Figure 4:**
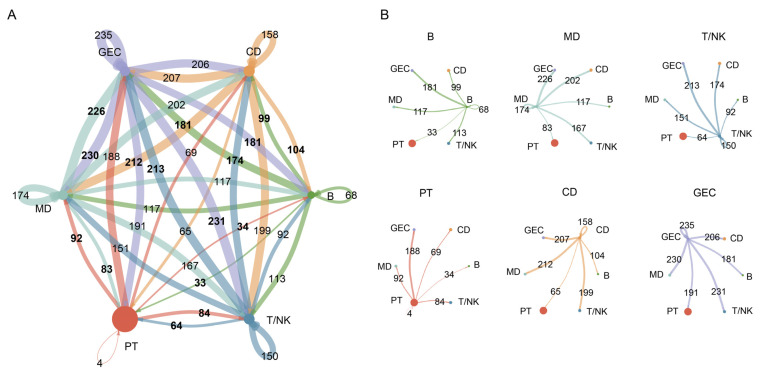
Cell–cell communication network in normal kidney immune microenvironment. (**A**) The number of interactions between any two cell types using a circle plot. The edge colors were consistent with the sources as senders, and edge weights were proportional to the interaction count. (**B**) Detailed view of the number of interactions sent from each cell type. Numbers indicated the quantity of ligand–receptor pairs for each intercellular link.

We used the CellChat 1.1.3 R package to calculate the aggregated cell–cell communication network by counting the number of links (Figure 4A) in BKPyV-uninfected kidneys. The circle plots were further used to show the number of interactions sent from each cell group (Figure 4B) in normal kidneys. The number of interactions sent from T/NK (B, MD) to PT, CD, and GEC was 64, 174, and 213 (B: 33, 99, and 181; MD: 83, 202, and 226), respectively. The cell–cell communication with immune cells (T/NK, B, and MD) was enhanced in GEC compared to PT and CD. PT had the smallest number of interactions compared with CD and GEC in BKPyV-uninfected kidneys. Together, in normal kidneys, PT had the smallest number of cell-cell communication interactions with immune cells, and GEC had the highest number, which indicated that PT were more suited for BKPyV replication than CD and GEC.

### 2.5. The Percentage of BKPyV Late Transcripts (VP1-3 and Agnoprotein) in BKPyV-Infected Kidneys

**Figure 5:**
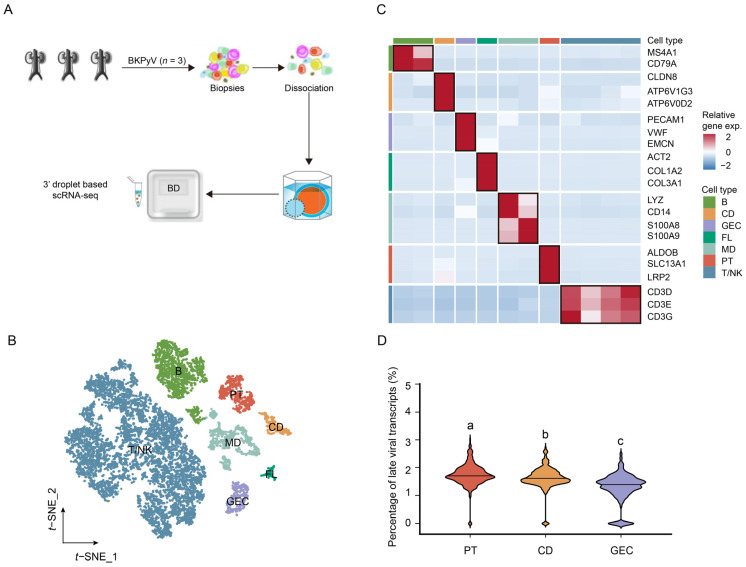
The percentage of BKPyV late transcripts (VP1−3 and agnoprotein) in BKPyV−infected kidneys (**A**) Overview of sample collection and single−cell transcriptomic analysis in BKPyV−infected kidneys (*n* = 3). (**B**) The t-SNE plot of 10,929 cells in BKPyV−infected kidneys color-coded by the major cell types as indicated. (**C**) The heatmap of relative expression map of known marker genes associated with each cell type. Relative expression values are scaled using mean−centering and transformed to a scale from −2 to 2. (**D**) The percentage of BKPyV late transcripts (VP1-3 and agnoprotein). Significant differences among PT, CD, and GEC were denoted by different letters (*p* < 0.05; LSD, ANOVA).

After quality control (cells with >500 genes and <4000 genes; and <30% of mitochondrial gene expression in UMI counts) and doublet removal, a total of 10,929 high-quality cells were further analyzed in BKPyV-infected kidneys. The average number of cells per sample was 3643, which met the needs for follow-up analysis based on the statistical power analysis (Supplementary Figure S1 and Table S1). We obtained scRNA-seq data from three BKPyV-infected kidneys (Figure 5A) to elucidate the cellular landscape. Then, we identified seven major cell types of BKPyV-infected kidneys using marker-based annotations (Figure 5B): PT (SLC13A1+, ALDOB+, and LRP2+); CD (CLDN8+, ATP6V1G3+, and ATP6V0D2+); GEC (EMCN+, VWF+, and PECAM1+); FL (COL1A2+, ACT2+, and COL3A1+); B (MS4A1+ and CD79A+); T/NK (CD3E+, CD3E+, and CD3G+); and MD (LYZ+, CD14+, S100A8+, and S100A9+) (Figure 5C). Then, we compared which cells were suited for BKPyV infection under infective conditions in terms of late viral transcripts (VP1-3 and agnoprotein). The rank of percentage of late viral transcripts was PT > CD > GEC. Different letters indicated statistically significant differences among the three groups (LSD, one-way ANOVA, *p* < 0.05) (Figure 5D). Additionally, previous studies showed that S phase of the cell cycle was beneficial to viral replication [28,29]. Therefore, we compared which cells were suited for BKPyV infection under infective conditions in terms of cell cycle. We found that the rank of percentage of late viral transcripts (VP1-3 and agnoprotein) was S phase > G2/M phase > G1 phase. Different letters indicated statistically significant differences among the three groups (LSD, one-way ANOVA, *p* < 0.05) (Supplementary Figure S3A). Moreover, the cell proportion of S phase in PT, CD, and GEC was 0.5, 0.3, and 0.2, respectively (Supplementary Figure S3B).

### 2.6. Complex Intercellular Communication Networks in BKPyV-Infected Kidneys

**Figure 6:**
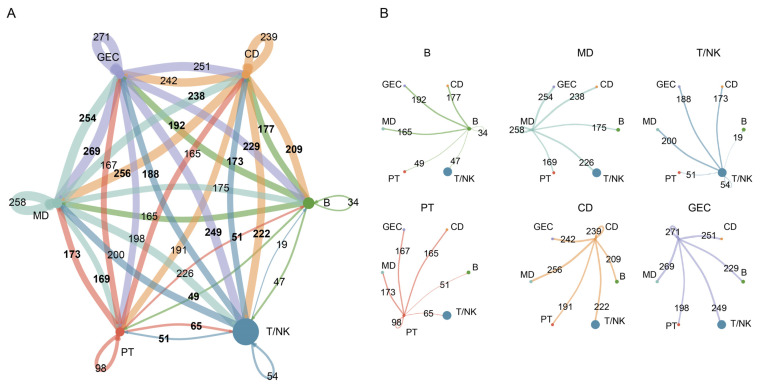
Cell–cell communication network in BKPyV-infected kidneys immune microenvironment. (**A**) The number of interactions between any two cell types using a circle plot. The edge colors were consistent with the sources as senders, and edge weights were proportional to the interaction count. (**B**) Detailed view of the number of interactions sent from each cell type. Numbers indicated the quantity of ligand–receptor pairs for each intercellular link.

We also used the CellChat 1.1.3 R package to calculate the aggregated cell-cell communication network by counting the number of links (Figure 6A) in BKPyV-infected kidneys. The circle plots were further used to show the number of interactions sent from each cell group (Figure 6B) in BKPyV-infected kidneys. The number of interactions sent from T/NK (B, MD) to PT, CD, and GEC was 51, 173, and 188 (B: 49, 177, and 192; MD: 169, 238, and 254), respectively. The cell–cell communication with immune cells (T, B, and MD) was enhanced in GEC compared to PT and CD. The PT had the smallest number of interactions compared with the CD and GEC in BKPyV-infected kidneys, which indicated that the PT were more suited for BKPyV replication than the CD and GEC.

### 2.7. Evolution of BKPyV Involvement in Kidneys to Indirectly Validate BKPyV Tropism

**Figure 7:**
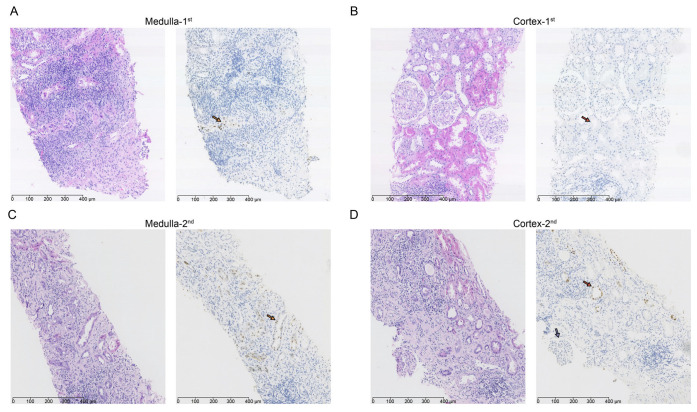
Evolution of BKPyV involvement to indirectly validate BKPyV tropism. The first biopsy (**A**,**B**) showed BKPyV only infected CD in the medulla ((**A**) left side: H&E staining 200×; right side: IHC staining of SV40T of the same area) (yellow arrow), while PT in the cortex was uninfected ((**B**) left side: H&E staining 200×; right side: IHC staining of SV40T of the same area) (red arrow). However, a repeated biopsy (3 months later) revealed that BKPyV had evolved from the medulla ((**C**) left side: H&E staining 200×; right side: IHC staining of SV40T of the same area) to PT in the cortex. The mesangial cells and endothelial cells within glomerular tufts were negative with anti-SV40T staining (purple arrow) ((**D**) left side: H&E staining 200×; right side: IHC staining of SV40T of the same area). Arrows with different color represent different cells. PT: proximal tubule cells (red arrow), CD: collecting duct cells (yellow arrow), GEC: glomerular endothelial cells (purple arrow).

To further verify the tropism of BKPyV to PT, we collected and compared the dynamic pathological findings of a KTR with progressive BKPyV infection at our institution. The first biopsy (Figure 7A,B) showed BKPyV only infected CD in the medulla (Figure 7A, left side: H&E staining 200×; right side: IHC staining of SV40T of the same area) (yellow arrow), while PT in the cortex was uninfected (Figure 7B, left side: H&E staining 200×; right side: IHC staining of SV40T of the same area) (red arrow). However, a repeated biopsy (3 months later) revealed that BKPyV had evolved from the medulla (Figure 7C, left side: H&E staining 200X; right side: IHC staining of SV40T of the same area) to PT in the cortex. The mesangial cells and endothelial cells within glomerular tufts were negative with anti-SV40T staining (purple arrow) (Figure 7D, left side: H&E staining 200×; right side: IHC staining of SV40T of the same area). These results indicated that BKPyV could spread from CD to PT, which suggested that the virus preferred PT to CD. Furthermore, BKPyV did not infect GEC when a large number of PTs were infected, showing that the virus preferred PT to GEC.

### 2.8. Potential Novel Target against BKPyV Infection

**Figure 8:**
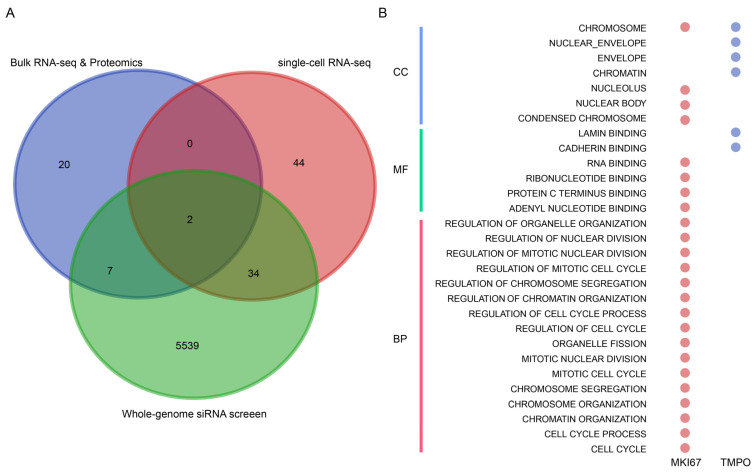
Identification of hub genes involved in BKPyV infection of PT. (**A**) Multi-omics to identify the hub genes in PT with BKPyV infection. Bulk RNA-seq and proteomics: identified the different expression genes that were altered in infected PT using bulk RNA-seq and quantitative proteomics [32]; single-cell RNA-seq: studied the genes covaried with BKPyV transcript levels via single-cell transcriptomic analysis [33]; whole-genome siRNA screen: explored essential genes for BKPyV infection in PT using the whole-genome RNA interference screen [34]. (**B**) The GO terms of MKI67 and TMPO.

Previous studies showed that BKPyV transmitted to PT was related to the worse outcome of renal allograft [30,31]. Together with previous results, we proposed that inhibition of BKPyV infection in PT based on the BKPyV tropism theory could help to improve the prognosis of KTRs with BKPyV infection. Then, we investigated the hub genes that affected the BKPyV preference for PT using multi-omics studies [32,33,34]. First, we obtained the different expression genes between the PT-uninfected group and PT-infected group via the bulk RNA-seq and proteome data from the previous study [32]. Second, we selected the genes whose levels covaried with the BKPyV late gene expression levels from the scRNA-seq data of Mock- and BKV-inoculated PT cells [33]. Third, we screened essential genes for BKPyV infection in PT using the whole-genome RNA interference screen [34]. Finally, we defined the sharing genes (MKI67 and TMPO) among multi-omics studies as the hub genes for BKPyV-infected PT (Figure 8A). The number of GO terms of TMPO was smaller than that of MKI67 (Figure 8B), which suggested the target of TMPO might cause fewer side effects. Then, we found that TMPO could be suggested as a novel target against BKPyV infection.

## 3. Discussion

In the present study, we defined that the BKPyV tropism-based ranking was PT, CD, and GEC using single-cell transcriptomic analysis of the cell-type signatures and immune microenvironment, which were indirectly demonstrated using clinical samples from repeat biopsies. Subsequently, the multi-omics analysis revealed that TMPO might serve as a hub gene for regulating BKPyV replication in PT.

We compared the BKPyV tropism among PT, CD, and GEC from three main cell signature factors: “essential TF for BKPyV infection”, “energy metabolism”, “the percentage of the late viral transcripts”, and “cell cycle.” First, it is well known that TFs play a crucial role in maintaining cell identity [35]. Furthermore, TFs specific to cell type could regulate viral gene expression and determine whether infection can be successfully established within a specific cell type [17].Additionally, the differentially expressed TFs were screened via an artificially set threshold compared with the TF activity, which could lead to erroneous results [36]. Therefore, the TF activity could generally play a more critical role in determining BKPyV infection in the kidneys than differentially expressed TFs. Thus, we quantified the cell-type specificity of TFs of PT, CD, and GEC based on the activity of TFs to identify which cell types were suitable for BKPyV infection. We found that P65 (also known as RELA or NFKB3) and CEBPB, which were reported to play crucial roles in regulating BKPyV gene expression [24,25], were PT-specific TFs according to the gene-TF regulatory networks (regulons) specificity score. The results indicated that BKPyV displayed a more significant preference for PT than CD and GEC. Second, a previous study suggested that BKPyV infection was generally related to enhanced cell metabolism due to the synthesis of many proteins and viral genome replication [37]. Therefore, by applying scMetabolism analysis to quantify the single-cell metabolic activity, we surprisingly found that PT displayed the highest glycolysis/gluconeogenesis metabolic activity, suggesting that PT were more beneficial for BKPyV infection than CD and GEC. The results were consistent with the electron microscope images of renal cells in that PT showed higher metabolism activities than CD and GEC under normal conditions. Third, the percentage of late BKPyV transcripts was significantly higher in PT than in CD and GEC (*p* < 0.05) in the infected kidneys, which suggested that PT were more suited for BKPyV infection than CD and GEC. The limitation of our study should be noted. The depth of BD scRNA-seq may not be sensitive enough for low levels of viral mRNAs. Fourth, previous studies reported that cell cycle was associated with BKPyV infection, which enabled viral genomes to be replicated [10,33]. Thus, we determined each cell cycle phase and found the cell proportion of S phase in PT were higher compared to CD and GEC, which suggested that PT were more suited for BKPyV replication than CD and GEC. We determined that the tropism-based ranking was PT, CD, and GEC in terms of cell signatures.

The immune microenvironment of the kidney is critical in determining the renal cell-type tropism of BKPyV based on previous studies [14,19]. Renal cell types with fewer interactions with immune cells could more easily escape immune system surveillance when infected by BKPyV. However, previous in vitro studies could not completely mimic the complex immune microenvironment of the kidney [20,38]. Therefore, their results were not consistent with the clinical observation in vivo. For example, they suggested that BKPyV in PT were spread from GEC in vivo based on their in vitro results [20,38]. However, previous studies reported that BKPyV could follow an ascending route of infection from CD to PT in vivo [18,39,40]. Moreover, in vivo, we also found that BKPyV spread from CD to PT and that GEC were rarely infected by BKPyV even when large numbers of PT were infected. Until recently, with the development of the scRNA-seq, it was possible to explore the cell-cell interaction between immune cells and different renal cells in vivo [23]. Therefore, we performed a cellular communication analysis based on the scRNA-seq data to identify the interplay between renal and immune cells. The number of interactions between PT and immune cells (T cells, B cells, and myeloid cells) was less than in CD and GEC, suggesting that BKPyV was more likely to escape immune surveillance in PT than in GEC and CD. We also speculated that GEC might not be susceptible to BKPyV infection, as GEC had the most frequent communications with immune cells. Consistently, the percentage of late viral transcripts in GEC was significantly lower than in PT and CD (*p* < 0.05). Together, we suggested that the tropism-based ranking was PT, followed by CD, and GEC from the perspective of the immune microenvironment.

We indirectly demonstrated the ranking of BKPyV tropism derived from scRNA-seq data by clinical observation of sequential biopsies, since in vitro models [19,20] do not entirely mimic the complex immune microenvironment of kidneys in vivo and BKPyV displays a very narrow host range that precludes direct investigation of the pathogenesis of BKPyV infection in animal models [21]. The clinical observation of sequential biopsies indicated that BKPyV could spread from CD to PT, suggesting that the virus preferred PT to CD. Furthermore, BKPyV did not infect GEC when a large number of PT were infected, indicating that the virus preferred PT to GEC. The clinical observation was also consistent with previous studies that BKPyV could follow an ascending route of infection from CD to PT in vivo [18,39,40]. Taken together, the clinical observation indicated that BKPyV preferred PT over CD and GEC.

We proposed that inhibition of BKPyV-infected PT based on the BKPyV tropism theory could help improve the prognosis of KTRs with BKPyV infection. Consequently, our objective was to identify the hub genes during BKPyV infection in PT. Although TFs played a crucial role in BKPyV infection, drugs that targeted them might cause adverse effects due to their participation in several other biologic processes [41]. Therefore, we integrated the multi-omics data sets to robustly identify TMPO as the drug target during the BKPyV infection process in PT [32,33,34]. Previous studies also showed that TMPO was associated with replication and cell cycle control [42,43], which indicated that it could play an important role in BKPyV replication. However, experiments are still needed to verify the detailed mechanism of TMPO against BKPyV infection. Taken together, we used multi-omics methods to suggest TMPO as a novel target gene against BKPyV.

In summary, the results of our single-cell transcriptomic analysis demonstrated that PT were the most suitable renal parenchymal cells for BKPyV infection and replication. As the hub gene that affected BKPyV tropism in PTs, TMPO might be a novel therapeutic target for the clinical treatment of BKPyV infection and BKPyVAN.

## 4. Materials and Methods

### 4.1. Sample Collection

This study was performed in accordance with the tenets of the Declaration of Helsinki, and Institutional review board approval was obtained from the First Affiliated Hospital of Sun Yat-sen University (2021-103). Informed consent was obtained from all patients. Healthy adult kidney tissues (n = 10) and BKPyV-infected kidney tissues (n = 3) were collected for single-cell RNA sequencing (scRNA-seq). We listed the accession ID for each sample (Supplementary Table S3). Three experienced clinicians diagnosed all the samples to ensure reliability (Supplementary Table S4). We also employed the Single-Cell One-sided Probability Interactive Tool (SCOPIT) for power analysis to evaluate the number of cells needed to be captured in each sample [44].

### 4.2. scRNA-Seq and Data Processing

The tissues from kidney biopsies were processed as previously reported with modifications [45,46]. Briefly, samples were first minced and digested with an enzyme to obtain a single-cell suspension (details are in the Supplemental Material S1 and S2). The BD rhapsody whole-transcriptome assay-analysis pipeline was employed to obtain the raw gene expression matrices. The raw gene expression matrices were then filtered, normalized, and batch corrected using the Seurat 4.0.2 R package [47,48] and selected according to the following criteria: cells with >500 genes and <4000 genes; and <30% of mitochondrial gene expression in UMI counts (details are in the Supplemental Material S3 [33,47,49,50,51,52,53]). The main cell clusters were identified using a graph-based clustering approach with resolution = 0.2. In addition, the major cell types were annotated by the canonical marker genes reported in previous studies [45,46,54,55].

### 4.3. Quantifying the Cell-Specific TF of Normal Adult Renal Cells

The TF gene regulatory networks at single-cell resolution were constructed using pySCENIC 0.11.2, a Python implementation of the SCENIC pipeline [56]. Briefly, the gene-TF regulatory networks (regulons) were first inferred according to the correlations between the expression of genes across cells using the GRNBoost2 algorithm [57]. Second, a TF motif analysis was performed to select potential direct-binding targets based on the cisTARGET databases (https://resources.aertslab.org/cistarget/, accessed on 6 December 2022) [58]. Third, the activity of the regulons was quantified by calculating an enrichment score for the target genes within each regulon using cell enrichment scores for each regulon. Finally, we identified specific cell-type regulons according to the regulon specificity score (RSS), which was calculated using the Jensen–Shannon Divergence (JSD) algorithm [59].

RSS = 1 − $\sqrt{JSD\left(P^{R},P^{C}\right)}$,

$P^{R}\;$indicated the distribution of regulon activity scores,

$P^{R}$ = ($P_{1}^{R}$, ···,$P_{n}^{R}$), where $\sum_{i=1}^{n}P_{i}^{R}=1$,

n represented the total number of cells,

$P^{C}\;$represented the distribution of cell type,

$P^{C}$ = ($P_{1}^{C}$, ···, $P_{1}^{C}$), where $\sum_{i=1}^{n}P_{i}^{C}=1$,

JSD ($P^{R}$, $P^{C}$) = H($\frac{P^{R}+PC}{2}$) − $\frac{H\left(P^{R}\right)+H\left(P^{c}\right)}{2}$, H(P)= −$\Sigma P_{i}\log\left(P_{i}\right)$, where H(P) indicated the Shannon entropy of a probability distribution P.

### 4.4. Assessment of the Host Cell Signatures

We employed the scMetabolism 0.2.1 R package [60] to quantify metabolic activity at the single-cell level. Furthermore, we evaluated the late viral transcripts according to the proportions of unique molecular identifies that mapped to the late viral transcripts (VP1-3 and agnoprotein) relative to those that mapped to host cellular transcripts [33]. BKPyV genome (GenBank accession number NC_001538.1) was used for alignment (details are in the Supplemental Material S4). Moreover, we identified the cell-cycle phase of each cell by calculating cell-cycle phase scores based on canonical markers [47].

### 4.5. Transmission Electron Microscopy (TEM)

The ultrastructural analysis was analyzed using TEM based on the standard procedures to identify the cell state of the kidney. In brief, the needle biopsy samples were cut into approximately 1 mm^2^ pieces. The samples were fixed in 2.5% glutaraldehyde (NO: A17876, Ala Aesar) in 0.1 M cacodylate buffer (NO: P-3813, Sinopharm chemical reagent). Post-fixation was performed with 1% OsO4 (NO: 18456, TED PELLA). Subsequently, the tissues were dehydrated through a graded series of ethanol and acetone before embedding them in EPON. Ultrathin sections were stained with 2% uranyl acetate and observed under TEM (JEM 1400 PLUS, Japan Electron Optics Laboratory Co., Ltd., Tokyo, Japan).

### 4.6. Cell-Cell Communication Analysis

We employed CellChat 1.1.3 R package to quantitatively infer and analyze cell–cell interaction networks from scRNA-seq data based on the CellChatDB, which contains 1939 validated molecular interactions [61]. scRNA-seq data with cells annotated as PT, CD, GEC, B cells, T cells, and myeloid cells were created as CellChat objects for subsequent analyses. Then, the total numbers of interactions were calculated using the compare interactions function in CellChat with default CellChatDB and the function computeCommunProb set raw.use = TRUE.

### 4.7. Immunohistochemical Staining

According to a previous study [30], the BKPyV infection scope in the kidneys was evaluated using HE staining and immunohistochemical staining with anti-SV40 large T antigen monoclonal antibody (DP02, Oncogene Research Products). We evaluated the scope of BKPyV in the kidneys by observing the results of two needle punctures. The patients diagnosed with BKPyV infection in CD from September 2020 to September 2021 at the First Affiliated Hospital of Sun Yat-sen University were enrolled for further analysis, which was considered the early phase of BKPyV infection. After a treatment period, we observed the infected sites in the kidney using secondary assays of the same patients to explore the range of BKPyV infection.

### 4.8. Predicting Potential Antiviral Targets

We integrated multi-omics studies to identify the hub genes involved in the BKPyV-infected PT, we integrated multi-omics studies. More detailed descriptions are given below. Justice et al. identified the hub genes altered in infected PT using quantitative proteomics and bulk RNA-seq [32]. An et al. reported 80 PT-related genes whose mRNA levels covaried with BKPyV transcript levels through single-cell transcriptomics analysis [33]. Furthermore, Zhao et al. studied the essential genes for BKPyV infection in PT using the whole-genome RNA interference screen [34]. Finally, we defined the sharing genes as the hub genes during BKPyV-infected PT.

## Data Availability

The scRNA-seq data in the study has been uploaded to the National Genomics Data Center (accession number HRA002472, https://ngdc.cncb.ac.cn/gsa-human/s/dO6YVnVb, 16 September 2022).

## Supplementary Materials

The following supporting information can be downloaded at: https://www.mdpi.com/article/10.3390/ijms24021330/s1.
