# Supplementary material for: Single-Cell Transcriptome Identifies the Renal Cell Type Tropism of Human BK Polyomavirus
Source: Int J Mol Sci. 2023 Jan 10;24(2):1330. doi: 10.3390/ijms24021330 (PMC9861348; doi:10.3390/ijms24021330)
Supplement: Supplementary file 1 [file ijms-24-01330-s001.zip › ijms-2135005-supplementary/ijms-2135005-supplementary.pdf]

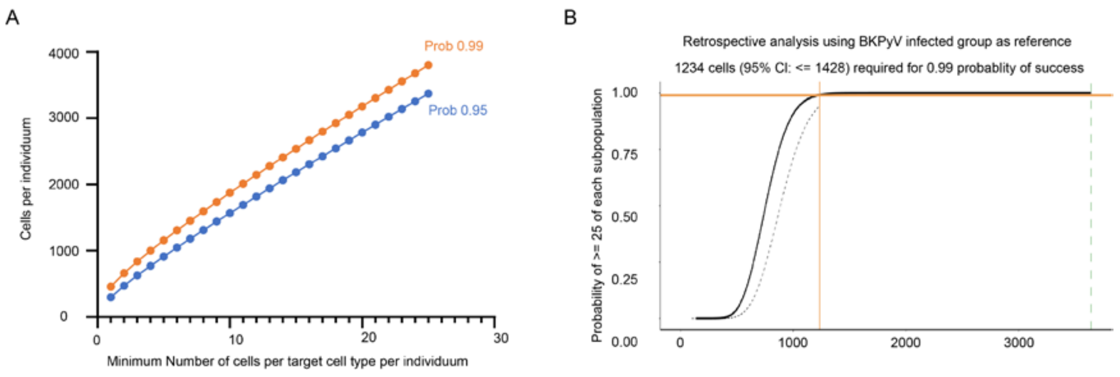

Figure S1. Power analysis on scRNA-seq sample size.

(A) Statistical power analysis performed using SCOPIT using both prospective and retrospective mode. Graph shows total number of cells in each individual sample (Cells per individuum) required to the minimum number of target cells in a rare cell type (population frequency 0.01 or 1%) in 1 cluster with either 0.99 or 0.95 probability of capture. **(B)** Graph shows the probability of capturing greater than or equal to 20 cells of a rare population with probability of 0.99 with dotted lines showing 95% confidence interval.

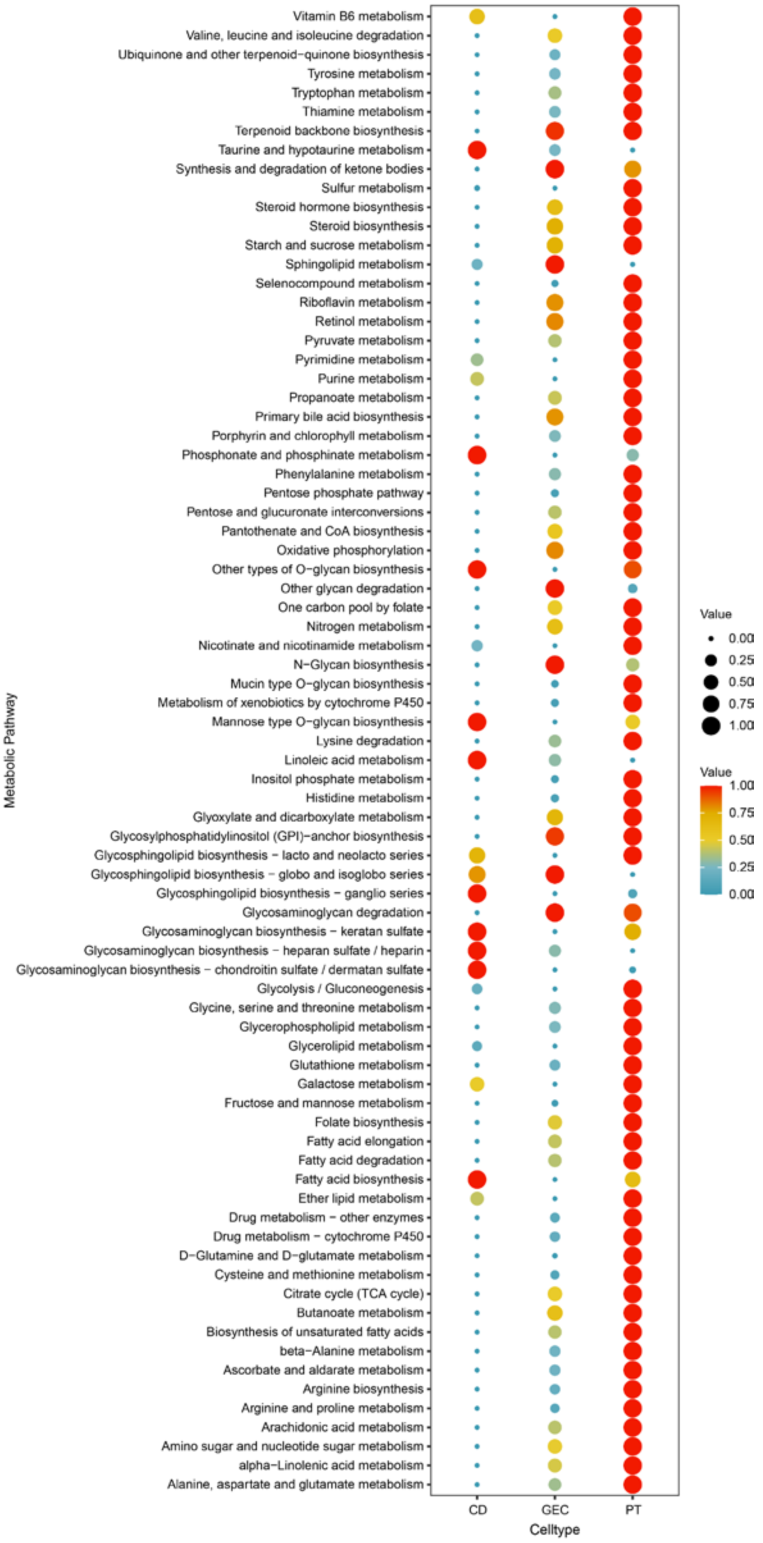

Figure S2. Comparison of the activity of metabolism among the normal renal cells.

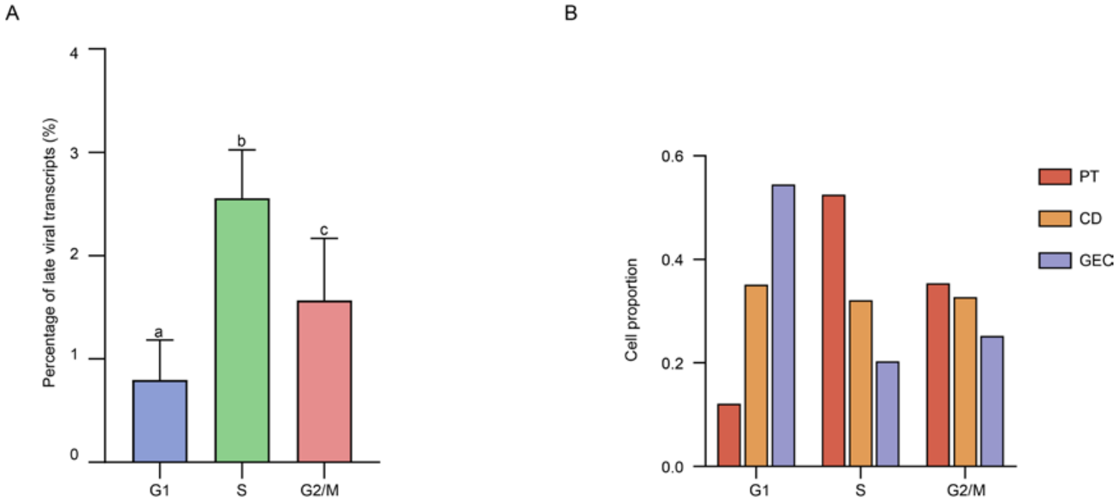

Figure S3. The relationship between cell cycle and BKPyV infection levels.

(A) The percentage of BKPyV late transcripts (VP1-3 and agnoprotein) among different stages of the cell cycle (G1, S, or G2/M). Significant differences among PT, CD, and GEC were denoted by different letters ($p < 0.05$; LSD, ANOVA).

(B) The cell proportion of different stages of the cell cycle phase among PT, CD, and GEC.

Table S1. The results of prospective and retrospective power analysis, and the medium number of cells per group.

Sample	Prospective power analysis	Seurat QC filtration	Retrospective power analysis
Healthy kidney group	Expect to detect 20 cells for frequency 0.01 when sequencing 2784-3179 cells	3857	Probability of detecting >= 20 cells of each subpopulation when at least 2054 cells (95% Cl: 2587) reguired for 0.99 probablility of success
BKPyV infected group		3643	

Table S3. The accession ID for each sample.

ID	Group	Accession ID
CSU1	Normal tissue	GSE171458
CSU1	Normal tissue	GSE171458
GXU1	Normal tissue	GSE131685
GXU2	Normal tissue	GSE131685
GXU3	Normal tissue	GSE131685
HCL1	Normal tissue	GSE195790
HCL2	Normal tissue	GSE195790
HCL3	Normal tissue	GSE195790
SYSU1	Normal tissue	HRA002472
SYSU2	Normal tissue	HRA002472
BKPyV1	BKPyV	HRA002472
BKPyV2	BKPyV	HRA002472
BKPyV3	BKPyV	HRA002472

Table S4. The information of patients.

ID	Sample	Age/Sex	Histology	BK viremia (copies/mL)	Maintenance immunosuppressive regimen	Source
BKPyV1	BKPyV	50/female	BKPyVAN Stage B3	9650	Mycolic acid +tacrolimas+ steroid	procedural paracentesis
BKPyV2	BKPyV	38/male	BKPyVAN Stage B1	72800	Mycolic acid +tacrolimas+ steroid	procedural paracentesis
BKPyV3	BKPyV	41/male	BKPyVAN Stage B1	2000	Mycolic acid +tacrolimas+ steroid	procedural paracentesis
Normal1	Normal	36/female	Negative	0	Mycolic acid +tacrolimas+ steroid	procedural paracentesis
Normal2	Normal	27/male	Negative	0	Mycolic acid +tacrolimas+ steroid	procedural paracentesis
Normal3	Normal	55/NA	Adjacent normal tissue	NA	NA	paracancerous tissues
Normal4	Normal	42/NA	Adjacent normal tissue	NA	NA	paracancerous tissues
Normal5	Normal	57/male	Adjacent normal tissue	NA	NA	paracancerous tissues

Normal6	Normal	59/female	Adjacent normal tissue	NA	NA	paracancerous tissues
Normal7	Normal	65/male	Adjacent normal tissue	NA	NA	paracancerous tissues
Normal8	Normal	66/male	Adjacent normal tissue	NA	NA	paracancerous tissues
Normal9	Normal	41/male	Adjacent normal tissue	NA	NA	paracancerous tissues
Normal10	Normal	57/male	Adjacent normal tissue	NA	NA	paracancerous tissues

Supplemental material S1. Single-cell Dissociation

Single-cell RNA-seq experiment was performed by experimental personnel in the laboratory of NovelBio Bio-Pharm Technology Co.,Ltd. The tissues of renal biopsy were surgically removed and kept in MACS Tissue Storage Solution (NO: 130-100-008, Miltenyi Biotec) until processing. The tissue samples were processed as described below. Briefly, samples were first washed with phosphate-buffered saline (PBS), minced into small pieces (approximately 1mm^3) on ice and enzymatically digested with 200 U/mL collagenase I (Worthington),50 U/mL collagenase IV (Worthington)and 30 U/mL DNase I (Worthington) for 25 min at 37°C, with agitation. After digestion, samples were sieved through a 40μm cell strainer, and centrifuged at 500g for 8 min. After the supernatant was removed, the pelleted cells were suspended in red blood cell lysis buffer (NO: 130-094-183, Miltenyi Biotec) to lyse red blood cells. After washing with RPMI-1640(Gibco), the cell pellets were re-suspended in sample buffer (BD Biosciences). Dissociated single cells were then stained for viability assessment using Calcein-AM (NO: C1430, Thermo Fisher Scientific) and Draq7 (NO: 564904, BD Biosciences).

Supplemental material S2. Single-cell RNA Sequencing

BD Rhapsody system was used to capture the transcriptomic information of the single cells. Single-cell capture was achieved by random distribution of a single-cell suspension across >200,000 microwells through a limited dilution approach. Beads with oligonucleotide barcodes were added to saturation so that a bead was paired with a cell in a microwell. The cells were lysed in the microwell to hybridize mRNA molecules to

barcoded capture oligos on the beads. Beads were collected into a single tube for reverse transcription and ExoI digestion. Upon cDNA synthesis, each cDNA molecule was tagged on the 5′ end (that is, the 3′ end of a mRNA transcript) with a unique molecular identifier (UMI) and cell barcode indicating its cell of origin. Whole transcriptome libraries were prepared using the BD Rhapsody single-cell whole-transcriptome amplification (WTA) workflow including random priming and extension (RPE), RPE amplification PCR and WTA index PCR. The libraries were quantified using a High Sensitivity DNA chip (Agilent) on a Bioanalyzer 2200 and the Qubit High Sensitivity DNA assay (Thermo Fisher Scientific). Sequencing was performed by illumina sequencer (Illumina, San Diego, CA) on a 150 bp paired-end run.

Supplemental material S3. Single-cell RNA Statistical Analysis

scRNA-seq data analysis was performed by NovelBio Bio-Pharm Technology Co.,Ltd. with NovelBrain Cloud Analysis Platform. We applied fastp [1] with default parameter filtering the adaptor sequence and removed the low quality reads to achieve the clean data. UMI-tools [2] was applied for Single Cell Transcriptome Analysis to identify the cell barcode whitelist. The UMI-based clean data was mapped to human genome (Ensemble version 100) utilizing STAR [3] mapping with customized parameter from UMI-tools standard pipeline to obtain the UMIs counts of each sample. Cells with >500 genes and <4,000 genes; and <30% of mitochondrial gene expression in UMI counts passed the cell quality filtering and mitochondria genes were removed in the expression table. Seurat 4.0.2 R package [4] was used for cell normalization and regression based on the expression table according to

the UMI counts of each sample and percent of mitochondria rate to obtain the scaled data. Moreover, for batch correction, we performed canonical correlation analysis using a diagonal implementation of CCA in Seurat 4.0.2 with 2000 variable genes [5]. PCA was constructed based on the scaled data with top 2000 high variable genes and top 20 principals were used for tSNE construction and UMAP construction.

Utilizing graph-based cluster method (resolution = 0.2), we acquired the unsupervised cell cluster result based the PCA top 10 principal and we calculated the marker genes by FindAllMarkers function with wilcox rank sum test algorithm under following criteria:1. lnFC > 0.25; 2. pvalue<0.05; 3. min.pct>0.1. In order to identify the cell type detailed, the clusters of same cell type were selected for tSNE analysis, graph-based clustering and marker analysis.

Supplemental material S4. The percentage of BKPyV late transcripts (VP1-3 and agnoprotein) in BKPyV infected kidenys

To profile host and BKPyV gene expression simultaneously, we constructed a hybrid reference genome containing both human (Ensemble version 100) and BKPyV (NCBI NC_001538.1) reference sequences. Paired-end reads from each single cell were aligned to the human-BKPyV hybrid genome using STAR 2.7.10a [3] with default parameters.41 Reads mapped to the human reference genome were used to quantify the gene expression levels of host genes. Reads mapped to the BKPyV genome were retrieved to quantify the viral gene expression levels of the virus using Cufflinks [6]. To attenuate the influence of any overlapping open reading frames of BKPyV genes on gene expression quantification,

we used gene-coding sequence (CDS) regions to define the transcripts of BKPyV genes in this study. Finally, the percentage of BKPyV late transcripts (VP1-3 and agnoprotein) were defined as follows [7]:

The percentage of BKPyV late transcripts (VP1-3 and agnoprotein) = ncounts (VP1-3 and agnoprotein) / ncounts (cell) * 100%.

References

1. Chen, S.; Zhou, Y.; Chen, Y.; Gu, J., fastp: an ultra-fast all-in-one FASTQ preprocessor. *Bioinformatics* **2018,** 34, (17), i884-i890.
2. Smith, T.; Heger, A.; Sudbery, I., UMI-tools: modeling sequencing errors in Unique Molecular Identifiers to improve quantification accuracy. *Genome Res* **2017,** 27, (3), 491-499.
3. Dobin, A.; Davis, C. A.; Schlesinger, F.; Drenkow, J.; Zaleski, C.; Jha, S.; Batut, P.; Chaisson, M.; Gingeras, T. R., STAR: ultrafast universal RNA-seq aligner. *Bioinformatics* **2013,** 29, (1), 15-21.
4. Hao, Y.; Hao, S.; Andersen-Nissen, E.; Mauck, W. M., 3rd; Zheng, S.; Butler, A.; Lee, M. J.; Wilk, A. J.; Darby, C.; Zager, M.; Hoffman, P.; Stoeckius, M.; Papalexi, E.; Mimitou, E. P.; Jain, J.; Srivastava, A.; Stuart, T.; Fleming, L. M.; Yeung, B.; Rogers, A. J.; McElrath, J. M.; Blish, C. A.; Gottardo, R.; Smibert, P.; Satija, R., Integrated analysis of multimodal single-cell data. *Cell* **2021,** 184, (13), 3573-3587 e29.
5. Butler, A.; Hoffman, P.; Smibert, P.; Papalexi, E.; Satija, R., Integrating single-cell transcriptomic data across different conditions, technologies, and species. *Nat Biotechnol* **2018,** 36, (5), 411-420.
6. Roberts, A.; Trapnell, C.; Donaghey, J.; Rinn, J. L.; Pachter, L., Improving RNA-Seq expression estimates by correcting for fragment bias. *Genome Biol* **2011,** 12, (3), R22.
7. An, P.; Cantalupo, P. G.; Zheng, W.; Saenz-Robles, M. T.; Duray, A. M.; Weitz, D.; Pipas, J. M., Single-Cell Transcriptomics Reveals a Heterogeneous Cellular Response to BK Virus Infection. *J Virol* **2021,** 95, (6).
